# Supplementary material for: Proton Beam Therapy versus Photon Radiotherapy for Stage I Non-Small Cell Lung Cancer
Source: Cancers (Basel). 2022 Jul 26;14(15):3627. doi: 10.3390/cancers14153627 (PMC9329768; doi:10.3390/cancers14153627)
Supplement: Supplementary file 1 [file cancers-14-03627-s001.zip › cancers-1806005-supplementary.pdf]

Supplementary Table S1. Prescribed radiation doses according to tumor location and radiation modality.

Dose fractionation	BED_{10} * (CGE)	All, No. (%)(n = 289)	Peripheral, No. (%) (n = 56)	Close to chest wall, No. (%) (n = 157)	Central, No. (%) (n = 76)	Photon, No. (%) (n = 177)	Proton, No. (%) (n = 112)
3 CGE x 20	78	15 (5.2)		2 (1.3%)	13 (17.1%)	8 (4.5)	7 (6.2)
3.5 CGE x 20	81.6	1 (0.3)			1 (1.3%)	1 (0.6)	
4 CGE x 15	84	22(7.6)		7 (4.5%)	15 (19.7%)	13 (7.3)	9 (8.0)
3 CGE x 22	85.8	5(1.7)			5 (6.6%)	4 (2.3)	1 (0.9)
4 CGE x 16	89.6	2 (0.7)	1 (1.8%)		1 (1.3%)	1 (0.6)	1 (0.9)
6 CGE x 10	96	15 (5.2)		9 (5.7%)	6 (7.9%)	9 (5.1)	6 (5.4)
7.5 CGE x 8	105	2 (0.7)		2 (1.3%)		2 (1.1)	
12 CGE x 4	105.6	6 (2.1)		2 (1.3%)	4 (5.3%)	6 (3.4)	
12.5 CGE x 4	112.5	27 (9.3)		20 (12.7%)	7 (9.2%)	3 (1.7)	24 (21.4)
8 CGE x 8	115.2	25(8.7)	3 (5.4%)	13 (8.3%)	9 (11.8%)	9 (5.1)	16 (14.3)
7 CGE x 10	119	6 (2.1)		1 (0.6%)	5 (6.6%)	1 (0.6)	5 (4.5)
13 CGE x 4	119.6	2 (0.7)		2 (1.3%)		2 (1.1)	
10 CGE x 6	120	1 (0.3)		1 (0.6%)		1 (0.6)	
12 CGE x 5	132	1 (0.3)			1 (1.3%)	1 (0.6)	
14 CGE x 4	134.4	6 (2.1)	2 (3.6%)	4 (2.5%)		6 (3.4)	
15 CGE x 4	150	153(52.9)	50 (89.3%)	94 (59.9%)	9 (11.8%)	110 (62.1)	43 (38.4)

CGE, cobalt gray equivalent; BED, biologically equivalent dose. * Biologically equivalent dose using an α/β ratio of 10 Gy.

Supplementary Table S2. Prognostic factors for local progression-free survival and overall survival.

Local progression-free survival	Univariate analysis			Multivariate analysis		
Variables	HR	95% CI	*p* value	HR	95% CI	*p* value
Proton beam therapy (vs. photon radiotherapy)	1.640	0.651-0.431	0.294			
Age (continuous)	0.981	0.922-1.044	0.538			
Female (vs. male)	0.181	0.024-0.097	0.097	0.255	0.033-1.961	0.189
ECOG ≥2 (vs. 0, 1)	0.381	0.051-2.863	0.348			
Charlson comorbidity index ≥2 (vs. 0, 1)	1.713	0.663-4.423	0.266	2.006	0.760-5.295	0.160
Adenocarcinoma (vs. others)	0.126	0.017-0.947	0.044	0.159	0.021-1.214	0.076
T2a (vs. T1)	2.423	0.955-6.146	0.062	2.224	0.843-5.871	0.106
Central tumor location (vs. peripheral)	2.829	1.123-7.128	0.027			
BED_{10}* ≥125 CGE (vs. <125 CGE)	0.207	0.068-0.630	0.006	0.228	0.073-0.716	0.011
**Overall survival**	**Univariate analysis**			**Multivariate analysis**		
Variables	HR	95% CI	*p* value	HR	95% CI	*p* value
Proton beam therapy (vs. photon radiotherapy)	1.189	0.703-2.012	0.519			
Age (continuous)	0.991	0.956-1.027	0.621			
Female (vs. male)	0.331	0.141-0.776	0.011	0.310	0.130-0.729	0.007
ECOG ≥2 (vs. 0, 1)	1.695	0.896-3.206	0.105	1.771	0.919-3.413	0.088
Charlson comorbidity index ≥2 (vs. 0, 1)	1.363	0.807-2.302	0.246			
Adenocarcinoma (vs. others)	0.823	0.462-1.468	0.511			
T2a (vs. T1)	2.001	1.174-3.410	0.011	1.512	0.860-2.659	0.151
Central tumor location (vs. peripheral)	1.643	0.963-2.801	0.068			
BED_{10}* ≥125 CGE (vs. <125 CGE)	0.402	0.231-0.699	0.001	0.426	0.240-0.756	0.004

HR, hazard ratio; CI, confidence interval; ECOG, Eastern Cooperative Oncology Group; BED, biologically equivalent dose; CGE, cobalt gray equivalent. * Biologically equivalent dose using an α/β ratio of 10 Gy.

Supplementary Table S3. Characteristics of patients for propensity matched analysis

Characteristics	Photon, No. (%) (n = 93)	Proton, No. (%) (n = 93)	*p* value	Standardized mean difference (%)
Age (median, IQR), years *	74 (69-80)	75 (69-80)	0.836	0.28
Female	18 (19.4)	17 (18.3)	1.000	1.08
ECOG performance status ≥2	8 (8.6)	6 (6.5)	0.781	2.15
Charlson comorbidity index ≥2	49 (52.7)	50 (53.8)	1.000	1.08
T2a stage (vs. T1)	29 (31.2)	30 (32.3)	1.000	1.08
Adenocarcinoma (vs. others)	28 (30.1)	31 (33.3)	0.753	3.23
BED10 † ≥125 CGE	44 (47.3)	43 (46.2)	1.000	1.08
Central tumor location (vs. peripheral)	31 (33.3)	33 (35.5)	0.877	2.15

IQR, interquartile range; ECOG, Eastern Cooperative Oncology Group; BED, biologically equivalent dose; CGE, cobalt gray equivalent; FEV1, forced expiratory volume in 1 second; DLCO, diffusion capacity for carbon monoxide. * Data are median (interquartile range). † Biologically equivalent dose using an α/β ratio of 10 Gy.

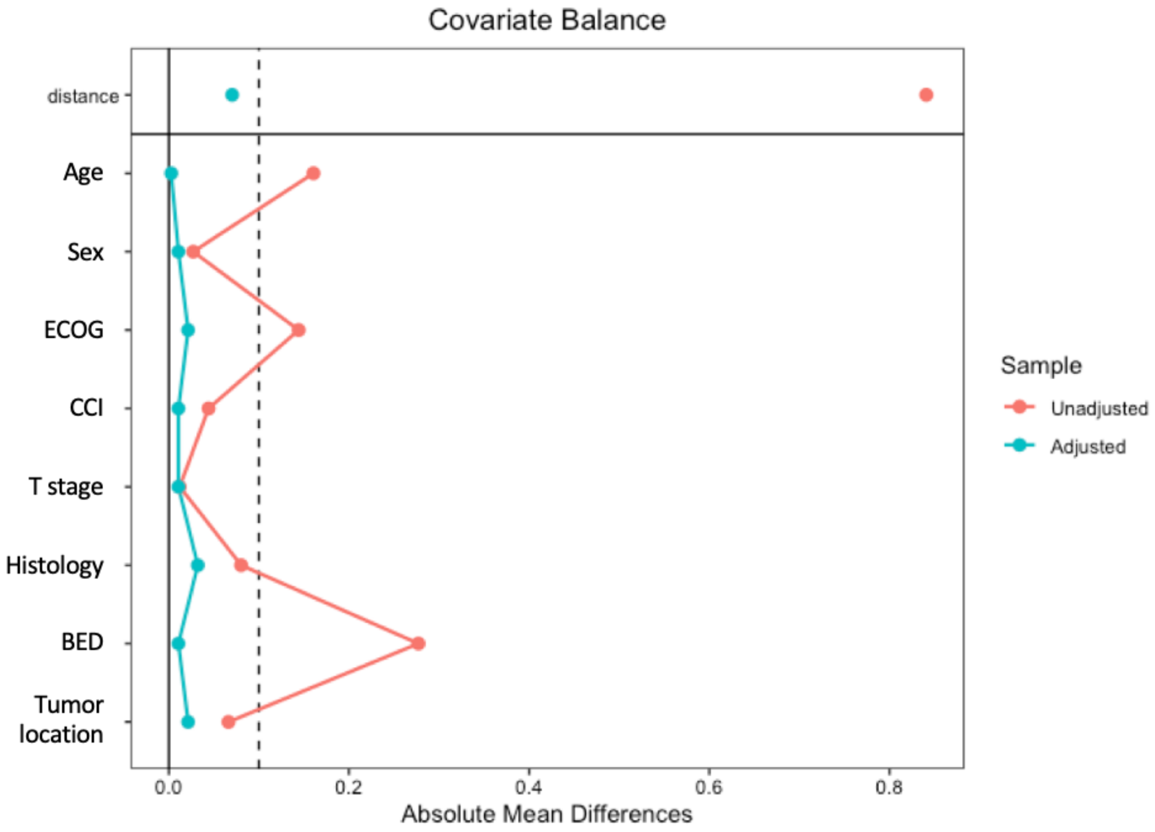

Supplementary Figure S1. Balance test using absolute mean differences before and after propensity-score matching. ECOG, Eastern Cooperative Oncology Group; CCI, Charlson comorbidity index; BED, biologically effective dose

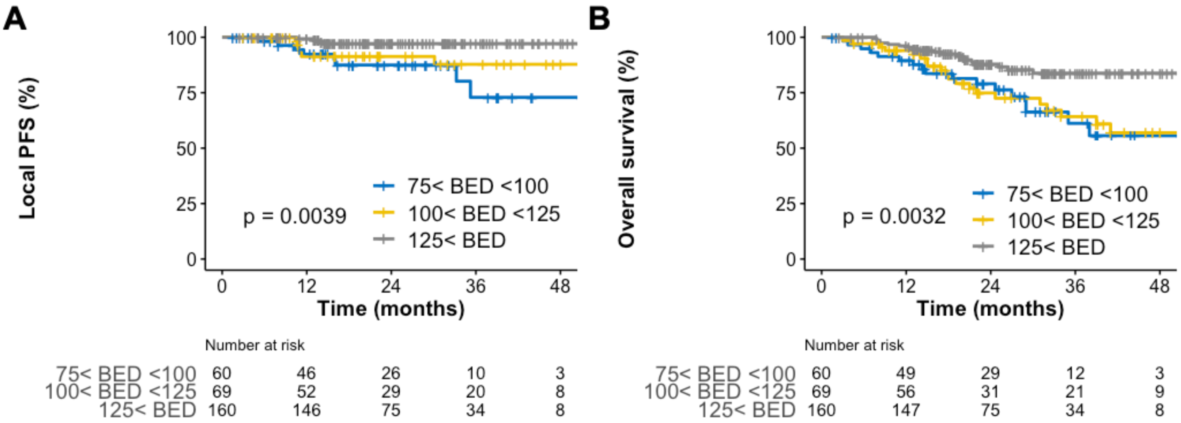

Supplementary Figure S2. Kaplan-Meier estimates of survival. (A) Local progression-free survival and (B) overall survival rates for all patients according to BED_{10} through 48 months. BED, biological equivalent dose; α/β ratio of 10 Gy was applied.
